# *In vitro* antimicrobial, antioxidant and cytotoxic properties of *Streptomyces lavendulae* strain SCA5

**DOI:** 10.1186/s12866-014-0291-6

**Published:** 2014-11-30

**Authors:** Pachaiyappan Saravana Kumar, Naif Abdullah Al-Dhabi, Veeramuthu Duraipandiyan, Chandrasekar Balachandran, Panthagani Praveen Kumar, Savarimuthu Ignacimuthu

**Affiliations:** Division of Microbiology, Entomology Research Institute, Loyola College, Chennai, 600 034 India; Department of Botany and Microbiology, Addiriyah Chair for Environmental Studies, College of Science, King Saud University, P.O. Box. 2455, Riyadh, 11451 Saudi Arabia; Department of Plant Biology and Biotechnology, Loyola College, Chennai, 600 034 India; Visiting Professor Programme, College of Science, Deanship of Scientific Research, King Saud University, Riyadh, Saudi Arabia

**Keywords:** SCA5, Antimicrobial, Antioxidant, Cytotoxicity

## Abstract

**Background:**

Actinomycetes are Gram-positive, often filamentous, bacteria known for their unsurpassed capacity for the production of secondary metabolites with diverse biological activities. The aim of the present study was to evaluate the antimicrobial, cytotoxic and antioxidant properties of *Streptomyces lavendulae* strain SCA5.

**Results:**

The ethyl acetate extract of SCA5 broth (EA-SCA5) showed antimicrobial activity with MIC value of 31.25 μg/ml. EA-SCA5 showed good antioxidant potential by scavenging 2, 2-diphenyl-picrylhydrazyl (DPPH) (IC_50_ 507.61 ± 0.66 μg/ml), hydroxyl radical (IC_50_ 617.84 ± 0.57 μg/ml), nitric oxide (IC_50_ 730.92 ± 0.81 μg/ml) and superoxide anion radical (IC_50_ 864.71 ± 1.15 μg/ml). The EA-SCA5 also showed strong suppressive effect on rat liver lipid peroxidation (IC_50_ 838.83 ± 1.18 μg/ml). The total phenolic content of SCA5 was 577.12 mg of GAE equivalents/gram extract. EA-SCA5 exhibited cytotoxic activity on A549 adenocarcinoma lung cancer cell line. It showed 84.9% activity at 500 μg/ml with IC_50_ value of 200 μg/ml. The gas chromatography mass spectrometry (GC-MS) analysis revealed the presence of one major bioactive compound actinomycin C2.

**Conclusions:**

The results of this study indicate that the EA-SCA5 could be probed further for isolating some medically useful compounds.

**Electronic supplementary material:**

The online version of this article (doi:10.1186/s12866-014-0291-6) contains supplementary material, which is available to authorized users.

## Background

Development of multiple drug resistance in microbes and tumour cells has become a major problem; there is a need to search for new and novel anticancer antibiotics [1]. Free radicals are known to be the major cause of various chronic and degenerative diseases, including aging, coronary heart disease, inflammation, stroke, diabetes mellitus and cancer. Oxidative stress occurs when there is excessive free radical production and/or low antioxidant defence, which leads to chemical alterations of biomolecules causing structural and functional modifications [2]. Currently available synthetic antioxidants show low solubility, promote negative health and have moderate antioxidant activity [3]. Over the past 75 years, natural compounds have led to the discovery of many drugs in the treatment of numerous human diseases [4]. By employing sophisticated techniques under various screening programs, the rate of discovery of natural compounds has exceeded 1 million so far [5]. Out of 22,500 biologically active compounds that have been extracted from microbes, 45% are from actinobacteria, 38% are from fungi and 17% are from unicellular bacteria [6]. Microbial secondary metabolites are one of the immense reservoirs of natural chemical diversity with potent biological activity [7]. Actinomycetes represent one of the most studied and exploited classes of bacteria for their ability to make a wide range of biologically active metabolites [8]. However, studies on microorganisms with respect to antioxidant and free radical scavenging activities are very limited. The studies on streptomycetes with respect to free radical scavenging activity are very few and most of the streptomycetes isolated are yet to be screened for bioactive secondary metabolites. The compounds isolated from marine *Streptomyces*, 2-allyoxyphenol and streptopyyrolidine, have been reported to possess antioxidant and no cytotoxic activity [9,10]. With today’s interest in new renewable sources of chemicals, polymers, anticancer, antioxidant and antimicrobial agents, the extracellular extract from soil microorganisms represent potential source to be explored. In this study, the antimicrobial, antioxidant and cytotoxic properties of extracellular crude extracts isolated from the fermented broth of soil-associated *Streptomyces lavendulae* strain SCA5 were investigated.

## Methods

### Isolation

The actinomycetes used in this work were isolated from soil samples collected from Vengodu (agricultural field), Thiruvannamalai district, Tamil Nadu, India (Latitude: 12°58′0033″, North; Longitude: 79° 70′5216″, East; Elevation ft/m 228.6/70.0). The actinomycetes isolation was carried out using the plating technique with serial dilution. Aliquots (0.1 ml) of 10^−2^, 10^−3^, 10^−4^, and 10^−5^ were spread on the starch casein agar (Himedia, Mumbai). To minimize the fungal and bacterial growth, actidione 20 mg/l and nalidixic acid 100 mg/l were added [11].

### Microbial organisms

The following Gram positive and Gram negative bacteria and fungi were used for the experiment. Gram positive: *Staphylococcus aureus* MTCC 96, *Micrococcus lutues* MTCC 106, *Bacillus subtilis* MTCC 441, *Staphylococcus epidermis* MTTC 3615, and Methicillin resistance *Staphylococcus aureus* (MRSA). Gram negative: *Klebsiella pneumoniae* MTCC 109, *Enterobacter aerogens* MTCC 111, *Vibrio parahaemolyticus* MTCC 450, *Yersinia enterocolitica* MTCC 840, *Salmonella typhimurium* MTCC 1251, *Shigella flexneri* MTCC 1457, *Proteus vulgaris* MTCC 1771, *Salmonella typhi-B* (SPB). Fungi: *Aspergillus flavus* (AF), *Botrytis cinerea* (BC), *Candida.krusei* (CK)*, Candida parapsilosis* (CP), *Malassesia pachydermatis* (MP), *Scopulariopsis sp*. (57), *Trichophyton mentagrophytes* (66), *Trichophyton rubrum* (101), *Candida albicans* (227), *Aspergillus niger* (1344). The reference bacterial cultures were obtained from the Institute of Microbial Technology (IMTECH), Chandigarh, India-160 036 and all the fungal cultures were obtained from the Department of Microbiology, Christian Medical College, Vellore, Tamil Nadu, India. Bacterial inoculums were prepared by growing cells in Mueller Hinton broth (MHB) (Hi-media) for 24 h at 37°C. The filamentous fungi were grown on Sabouraud dextrose agar (SDA) slants at 28°C for 10 days and the spores were collected using sterile double distilled water and homogenized. Yeast was grown on Sabouraud dextrose broth (SDA) at 28°C for 48 h.

### Cross streak method and media Optimization

The antimicrobial activity of actinomycetes isolates was performed by using cross streak method [12]. Antagonism was observed by the inhibition of test organism. *Streptomyces lavendulae* strain SCA5 was grown on the following media for the production of bioactive compounds in an orbital shaker (150 rpm at 30°C): Antibiotic production media (APM), Fermentation media (FEM), Glucose yeast extract malt media (GLM), M3 media, Modified nutrient glucose media (MNGA), M6 media and Yeast peptone glucose media (YPG). The culture was grown with continuous shaking on a rotary shaker (150 rpm) at 30°C for 10 days. The antimicrobial activity was tested for fermented broth against microbes using [13].

### Culture characterization

Cultural and morphological features of SCA5 were characterized by following [14]. Visual observation by light microscopy and Gram-staining were performed for further identification [15]. Biochemical reactions, different temperatures, NaCl concentration, pH level, pigment production and acid or gas production were done following the methods [16]. The total genomic DNA was extracted by using Hipura *Streptomyces* DNA spin kit-MB 527-20pr from Hi-media, according to the manufacturer’s protocol. The actinomycetes DNA fragments were amplified using Universal primers 16S rRNA and PCR reactions were standardized as follows: initial denaturation at 94°C for 3 min, followed by 35 cycles of 1 min at 94°C, 54°C for 1 min, 72°C for 2 min and a final extension at 72°C for 8–10 min, stop at 4°C for 1 h. The PCR products were stored at 4°C and visualized by electrophoresis. The gel was photographed in gel documentation system. The amplified product was purified and sequenced with two fragments of the 27F (5′AGT TTG ATC CTG GCT CAG 3′) and 1492R (5′ACG GCT ACC TTG TTA CGA CTT 3′) region in both the directions and the sequences obtained were submitted to Genbank. Phylogenetic tree was constructed using the neighbour-joining DNA distance algorithm using software MEGA (version 4.0) [17].

### Cultivation and extraction of antimicrobial metabolites from *Streptomyces lavendulae* strain SCA5

Well grown slant culture of the *Streptomyces lavendulae* strain SCA5 was used for the preparation of seed culture. The seed culture was inoculated in 50 ml medium containing the optimized production media and incubated for 10 days in a rotary shaker (150 rpm) at 30°C. The inoculums (10%) were transferred into 150 ml production medium in 250 ml Erlenmeyer flasks and kept for fermentation for ten days. After fermentation, the broth was filtered through blotting paper and the supernatant was separated. The supernatant was extracted twice with ethyl acetate. After separation, the organic phase was dried over Na_2_SO_4_ (anhydrous). The extract was then concentrated in a rotary vacuum. The crude extracts were stored at 4°C.

### Antibiogram of *Streptomyces lavendulae* strain SCA5

The antimicrobial activity of the ethyl acetate extract of SCA5 (EA-SCA5) was assayed using the standard Kirby-Bauer disc diffusion method [18]. Petri plates were prepared with 20 ml of sterile Mueller Hinton agar (MHA) (Hi-media, Mumbai). The test cultures were swabbed on the top of the solidified media and allowed to dry for 10 min. The tests were conducted at 2.5 mg/disc concentrations of EA-SCA5. The loaded discs were placed on the surface of the medium and left for 30 min at room temperature for compound diffusion. Negative control was prepared using respective solvent (DMSO). Streptomycin (25 μg/disc) for bacteria and Ketocanozole (30 μg/disc) for fungi was used as positive controls. The plates were incubated over night at 37°C for bacteria and at 28°C for fungi and the zones of inhibition were recorded. Diameters of the zones of inhibition were measured using a zone scale from Hi-media and expressed in millimetres. When the zone of inhibition was 0 to 4 mm it was considered weak activity; when the zone of inhibition was 5 to 10 mm it was considered moderate activity; when the zone of inhibition was 11 to 15 mm it was considered good activity.

### Minimal inhibitory concentrations of EA-SCA5

The minimal inhibitory concentrations (MICs) of EA-SCA5, defined as the lowest concentration in the micro titter plate with no growth (i.e., no turbidity) of the inoculated microorganism, was carried out as described by the Clinical and Laboratory Standards Institute (CLSI, 2005) with slight modification [19]. The EA-SCA5 was dissolved in DMSO in a concentration of 1000 μg/ml. The serial two fold dilutions of the EA-SCA5 (1000, 500, 250, 125, 62.5, 31.2, and 15.6 μg/ml) were prepared for MIC tests. MIC tests were carried out in Muller–Hinton Broth for bacteria and Sabouraud dextrose broth for fungi; the organisms were added to 96 well micro titter plate containing 0.1 ml broth. The 3 μl of log phase culture was introduced into respective wells and the final inoculum size was 1×10^5^ cfu/ml. The plates were incubated at 37°C for bacteria and 28°C for fungi. Streptomycin for bacteria and Ketocanozole for fungi were used as positive controls. Negative (water) and solvent controls (DMSO) were also included. 5 μl of the test broth was introduced on plain Mueller Hinton agar for bacteria and Sabouraud dextrose agar plates for fungi to observe the viability of the organism. MIC was determined as the lowest concentration which inhibited complete growth.

### Antioxidant properties

#### DPPH radical scavenging assay

DPPH quenching ability of EA-SCA5 was measured according to [20]. A methanol DPPH solution (0.15%) was mixed with serial dilutions (200–1,000 μg/ml) of EA-SCA5 and after 10 min, the absorbance was read at 515 nm. The radical scavenging activity was expressed as IC_50_ (μg/ml), (the dose required to cause a 50% inhibition). Vitamin C was used as standard. The ability to scavenge the DPPH radical was calculated by the following formula:

1 $$ \mathrm{DPPH}\ \mathrm{radical}\ \mathrm{scavenging}\ \mathrm{activity}\ \%=\left({\mathrm{A}}_0\hbox{--} {\mathrm{A}}_1\right)\mathrm{A}0\times 100 $$

Where A_0_ is the absorbance of the control at 30 min and A1 is the absorbance of the sample at 30 min. All samples were analyzed in triplicate.

### Determination of hydroxyl radical scavenging activity

The hydroxyl radical scavenging assay was performed as described by the method of [21] with minor changes. All solutions were prepared freshly. Stock solutions of EDTA (1 mM), FeCl_3_ (10 mM), ascorbic acid (1 mM), H_2_O_2_ (10 mM) and deoxyribose (10 mM) were prepared in distilled deionized water. The assay was performed by adding 0.1 ml EDTA, 0.01 ml of FeCl_3_, 0.1 ml of H_2_O_2_, 0.36 ml of deoxyribose, 1.0 ml of extract (200–1,000 μg/ml) each dissolved in distilled water, 0.33 ml of phosphate buffer (50 mM, pH 7.4) and 0.1 ml of ascorbic acid in sequence. The mixture was then incubated at 37°C for 1 h. About 1.0 ml portion of the incubated mixture was mixed with 1.0 ml of (10%) TCA and 1.0 ml of (0.5%) TBA (in 0.025 M NaOH containing 0.025 M NaOH BHA) to develop the pink chromogen and read at 532 nm. The hydroxyl radical scavenging activity of the extract was reported as the percentage of inhibition of deoxyribose degradation and was calculated according to the formula (1).

### Nitric oxide radical inhibition assay

Sodium nitroprusside in aqueous solution at physiological pH spontaneously generates nitric oxide; it interacts with oxygen to produce nitrite ions, which can be estimated by the use of Griess Illosvoy reaction [22]. In the present investigation, Griess Illosvoy reagent was modified using naphthylethylenediamine dihydrochloride (0.1% w/v) instead of 1-naphthylamine (5%). The reaction mixture (3 ml) containing sodium nitroprusside (10 mM, 2 ml), phosphate buffer saline (0.5 ml) and EA-SCA5 (200–1000 μg/ml) or standard solution (0.5 ml) was incubated at 25°C for 150 min. After incubation, 0.5 ml of the reaction mixture containing nitrite was pipetted and mixed with 1 ml of sulphanilic acid reagent (0.33% in 20% glacial acetic acid) and allowed to stand for 5 min for completing diazotization. Then, 1 ml of naphthylethylenediamine dihydrochloride (1%) was added, mixed and allowed to stand for 30 min. A pink colored chromophore was formed in diffused light. The absorbance of these solutions was measured at 540 nm against the corresponding blank. Vitamin C was used as standard. The scavenging activity was calculated using the formula (1).

### Superoxide scavenging activity

Superoxide scavenging activity of EA-SCA5 was determined by monitoring the competition of those with NBT for the superoxide anion generated by the PMS–NADH system [23]. Superoxide radicals were generated in 1 ml of 20 mM Tris–HCl buffer pH 8.0 containing 0.05 mM nitroblue tetrazolium (NBT), 0.01 mM phenazine methosulphate (PMS), and different concentrations (200–1,000 μg/ml) of EA-SCA5 were pre-incubated for 2 min. The reaction was initiated by the addition of 0.078 mM NADH. Blue chromogen, formed due to NBT reduction, was read at 560 nm. Results were expressed as percentage of inhibition of superoxide radicals.

### Animals

Male albino Wistar rats bred in the animal house of Entomology Research Institute, weighing 170 ± 5 g were used in the studies. The animals were kept in polypropylene cages, under controlled temperature, humidity and 12/12 light/dark cycles. The animals were fed pellet diet (Pranav Agro Industries Ltd., Maharashtra) and water ad libitum. This study was carried out with prior approval from Institutional Animal Ethical Committee (IAEC-ERI-LC-04/13).

### Inhibition of lipid peroxidation in rat liver homogenate

The inhibition effect of EA-SCA5 on lipid peroxidation was determined according to the thiobarbituric acid method [24]. FeCl_2_–H_2_O_2_ was used to induce liver homogenate peroxidation. In this method, 0.2 ml of EA-SCA5 extract (200–1000 μg/ml) was mixed with 1 ml of 1% liver homogenate (each 100 ml homogenate solution contains 1 g rat liver); then 50 μl of FeCl_2_ (0.5 mM) and H_2_O_2_ (0.5 mM) was added. The mixture was incubated at 37°C for 60 min; then 1 ml of trichloroacetic acid (15%) with thiobarbituric acid (0.67%) was added and the mixture was heated in boiling water for 15 min. The absorbance was recorded at 532 nm. Vitamin C was used as positive control. The percentage of inhibition was calculated using the formula (1).

### Total phenolic content (TPC)

Total phenolic content of EA-SCA5 was determined according to the Folin-Ciocalteu spectrophotometric method with some modifications [25]. Briefly, 0.1 (200–1,000 μg/ml), EA-SCA5 was mixed with 1.9 ml of distilled water and 1 ml of diluted Folin-Ciocalteu’s phenol reagent and allowed to react for 5 min. Then, 1 ml of 100 g/l Na_2_CO_3_ solution was added. After 2 h of reaction at 25°C, the absorbance at 765 nm was determined. The sample was tested in triplicate and a calibration curve with six data points for gallic acid was obtained. The results were compared to gallic acid calibration curve and the total phenolic content was expressed as mg of gallic acid equivalents per gram of extract.

### Reducing power

The determination was carried out as described by Oktay, Gu lc_in, and Ku frevioglu [26]. Briefly, different concentrations of EA-SCA5 (200–1000 μg/ml) were mixed with phosphate buffer (2.5 ml, 0.2 mol/l, pH 6.6) and K_3_Fe (CN)_6_ (2.5 ml, 1%). The mixtures were incubated for 20 min at 50°C. A portion (2.5 ml) of trichloroacetic acid solution (10%) was added to the mixture, which was then centrifuged at 10 000 g for 10 min. The upper layer of solution (2.5 ml) was mixed with deionized water (2.5 ml) and FeCl_3_ (0.5 ml, 0.1%), and the absorbance was measured at 700 nm and was compared with standard BHT absorbance.

### Cell line maintenance and growth conditions

A549 adenocarcinoma lung cancer cell line was obtained from National Institute of Cell Sciences, Pune and was maintained in complete tissue culture medium DMEM (Dulbecco’s modified eagle’s medium) with 10% Fetal Bovine Serum and 2 mM L-Glutamine, along with antibiotics (about 100 IU/ml of penicillin, 100 μg/ml of streptomycin) with the pH adjusted to 7.2. The cell lines were maintained at 37°C at 5% CO_2_ in CO_2_ incubator [27]. Cultures were viewed using an inverted microscope to assess the degree of confluency and the absence of bacterial and fungal contaminants were confirmed.

### Cytotoxic properties

The cytotoxicity was determined according to the method with some changes [28]. Cells (5000 cells/well) were seeded in 96 well plates containing medium with different concentrations such as 100 μl, 75 μl, 50 μl, 25 μl, 12.5 μl and 6.25 μl. The cells were cultivated at 37°C with 5% CO_2_ and 95% air in 100% relative humidity. After various durations of cultivation, the solution in the medium was removed. An aliquot of 100 μl of medium containing 1 mg/ml of 3-(4, 5-dimethylthiazol-2-yl)-2, 5-diphenyl-tetrazolium bromide (MTT) was loaded to the plate. The cells were cultured for 4 h and then the solution in the medium was removed. An aliquot of 100 μl of DMSO was added to the plate, which was shaken until the crystals were dissolved. The cytotoxicity against cancer cells was determined by measuring the absorbance of the converted dye at 570 nm in an ELISA reader. Cytotoxicity of each sample was expressed as IC_50_ value. The IC_50_ value is the concentration of test sample that causes 50% inhibition of cell growth, averaged from three replicate experiments.

### TLC analysis

The TLC profile of the active extract was carried out on Merck silica gel 60F_254_ pre coated aluminium plates of layer thickness 0.2 mm developing system chloroform : methanol 4:1. The plates were viewed under normal white light and UV light at 366 nm. Derivatization was carried out by 10% alcoholic sulphuric acid reagent, heated at 110°C for 5 minutes and also by exposure to iodine vapours. Phenol was located by 0.1% alcoholic ferric chloride and quinone was located by 0.1% alcoholic NaOH.

### GC-MS analysis

The active ethyl acetate crude was subjected to GC-MS analysis on GC-MS- 5975 (AGILENT), column DB 5 ms Agilent, dimension length- 30.0 m, ID- 0.2 mm, flim thickness- 0.25 μm with temperature program- 70–300°C, 10°C/min, injection temperature- 240°C, carrier gas- helium, flowrate- 1.51 ml/min, equipped with GC-MS NIST-II library.

### Statistical analysis

The data for biochemical and physiological parameters were analyzed and expressed as means ± SEM. The IC_50_ values were calculated from linear regression analysis. Results were processed by computer program, Microsoft Excel (2007).

## Results

### Isolation, characterization and identification of antagonistic actinomycetes

Based on the colony morphology and stability in subculturing, 37 suspected *Streptomyces* cultures were purified on ISP-2 slants. The isolates were initially screened to determine their ability to produce antimicrobial compounds. In the initial screening 8% showed good activity, 27% showed moderate activity, 24% showed weak activity and 40% showed no antagonistic activity against fungi; similarly 10% showed good activity, 27% showed moderate activity, 32% showed weak activity and 30% showed no activity against bacteria. Among the strains tested, SCA5 showed good antimicrobial activity against the tested pathogens. So SCA5 strain was identified by morphological, biochemical, physiological characteristics and 16S rRNA amplification. Table 1 shows the morphological features of the strain. SCA5 grew on all media used and had a grey to white aerial mycelium and light to dark greyish white vegetative mycelium. Diffusible pigments were not observed on the media tested. The isolate produced moderately branching, non-fragmenting substrate hyphae. Aerial hyphae were formed on all media except Muller Hinton agar, Zobell marine agar and Sabouraud dextrose agar; moderate growth was observed on skim milk agar. Based on the morphological characteristics, SCA5 was tentatively attached to the genus *Streptomyces* sp*.* Table 2 shows the physiological properties of SCA5. Optimal growth of strain was observed at 30°C and at pH 7 and in the presence of NaCl in the range of 1–9% (very good growth). Strains were able to grow at 13% NaCl (moderate to good growth) and at 30°C (maximum temperature growth), but were unable to grow at pH 5 and pH3. SCA5 was able to produce enzymes such as amylase, chitinase, protease, gelatinase, and lipase. Further, DNA preparation and PCR amplification of SCA5 strain genomic DNA resulted in 1500 bp amplicon approximately. 16S rDNA sequence was determined and Blast analysis was performed, which confirmed that the isolate belonged to *Streptomyces lavendulae* (bases 1 to 1459 linear DNA). The strain SCA 5 had 100% similarity with *Streptomyces lavendulae subsp. lavendulae* (AB184731); SCA5 was submitted to the GenBank, NCBI under the accession number, KC315780; phylogenetic tree was constructed (data given as Additional file 1). Based on the antimicrobial activity the *Streptomyces lavendulae* strain SCA5 was taken to assess the cytotoxicity and antioxidant properties.

**Table 1:** **Morphological features of** ***Streptomyces lavendulae*** **strain SCA5 on different media**

**Media**	**Aerial mycelium**	**Substrate mycelium**	**Diffusible pigment**	**Reverse side**	**Growth**
AIA	Grey	Grey	-	White	+++
Agar	White	White	-	White	+
ISP 2	White	White	-	Brownish yellow	+++
MHA	-	Slimy yellow	-	Slimy yellow	+
SCA	Greyish White	Greyish white	-	Yellow	+++
SDA	-	Yellow	-	Yellow	++
SKM	Yellowish white	Yellowish white	-	Yellow	++
STP	Dark grey	Dark grey	-	Yellowish grey	+++
YPG	Whitish yellow	Yellow	-	Yellow	+++
ZMA	-	Slimy Yellow	-	Whitish yellow	+

+++ Good growth; ++ Moderate growth; + Weak growth: − absent.

**Table 2:** **Physiological and biochemical characteristics of** ***Streptomyces lavendulae*** **strain SCA5**

**Characteristics**	**Results**
Gram staining	Positive
Shape and growth	filamentous aerial growth
Range of temperature for growth	25°C to 37°C
Optimum temperature	30°C
Range of pH for growth	3 to 11
Optimum pH	7
Growth in the presence of NaCl	1 to 9%
Amylase	+++
Chitinase	+
Protease	++
Gelatinase	+++
Lipase	++

+++ Good activity; ++ Moderate activity; + Weak activity.

### Optimization of media, extraction and antibiogram

**Figure 1:**
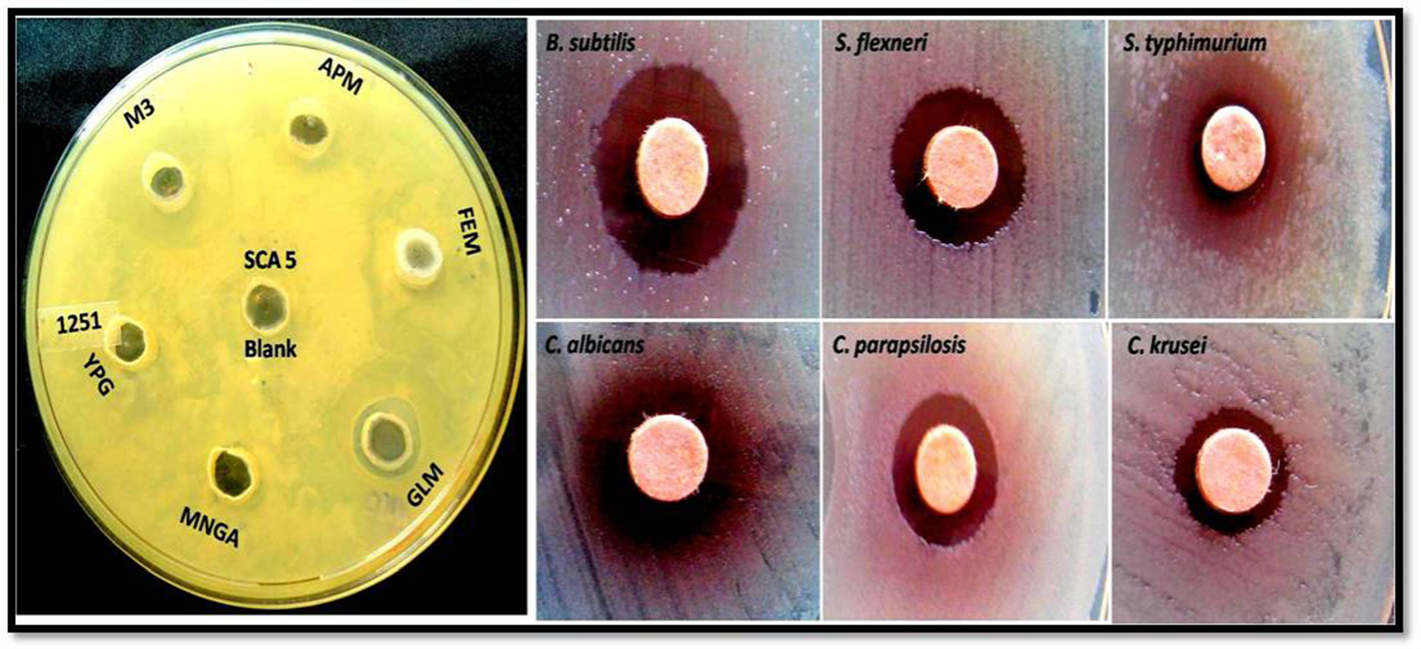
**Antimicrobial activity of EA-SCA5 using disc diffusion method.**

Of the various antibiotic production medium, M3 medium was highly efficient for *Streptomyces lavendulae* strain SCA5; the broth showed good activity against the tested pathogens. SCA5 was mass produced using optimized medium; the broth was extracted using ethyl acetate. The antimicrobial activities of EA-SCA5 are shown in Figure 1. EA-SCA5 showed good activity against 13 bacterial and 10 fungal pathogens with zones of inhibition ranging from 10 to 15 mm for bacteria and 10–13 mm for fungi (Table 3) [29].

**Table 3:** **Antimicrobial activity of** ***Streptomyces lavendulae*** **strain SCA5 ethyl acetate extract using disc diffusion method**

**Microbes**		
**Zone of inhibition (mm)**		
**EASCA5 (2.5 mg/disc)**		**Ketoconazole (30 μg/disc)**
*Aspergillus flavus*	12	20
*Aspergillus niger*	10	10
*Botrytis cinerea*	10	12
*Candida albicans*	13	15
*Candida.krusei*	11	16
*Candida parapsilosis*	11	35
*Malassesia pachydermatis*	10	24
*Scopulariopsis sp.*	12	19
*Trichophyton mentagrophytes*	13	13
*Trichophyton rubrum*	10	12
**Gram positive bacteria**		**Streptomycin (25 μg/disc)**
*Staphylococcus aureus*	13	27
*Micrococcus luteus*	15	30
*Bacillus subtilis*	11	23
*Staphylococcus epidermidis*	13	25
MRSA	12	24
**Gram negative bacteria**		
*Klebsiella pneumonia*	11	28
*Enterobacter aerogens*	10	24
*Vibrio parahaemolyticus*	10	18
*Yersinia enterocolitica*	10	20
*Salmonella typhimurium*	14	21
*Shigella flexneri*	15	29
*Proteus vulgaris*	10	18
*Salmonella typhi-B*	13	11

EA-SCA5: Ethyl acetate extract, Ketoconazole: Antifungal Agent.

Streptomycin: Antibacterial agent.

### *In vitro* antimicrobial assay (MIC)

The EA-SCA5 showed broad spectrum of activity against Gram positive and Gram negative bacteria with the MIC value of 125 μg/ml; the MIC value against fungi was 31.25 μg/ml (Table 4). EA-SCA5 showed significant activity against *M. lutues, S. flexneri* (MIC: 125 μg/ml), Methicillin resistant *S. aureus* (MRSA), *S. typhi-B* (SPB) (MIC: 250 μg/ml), *S. aureus, B. subtilis, S. epidermis, K. pneumonia* (MIC: 500 μg/ml), *E. aerogens, V. parahaemolyticus, Y. enterocolitica, S. typhimurium, P. vulgaris* (MIC: 1000 μg/ml). EA-SCA5 showed significant antifungal activity against *A. flavus* (MIC: 31.25 μg/ml), *M. pachydermatis, T. mentagrophyte* (MIC: 250 μg/ml), *C. parapsilosis*, *C. krusei, Scopulariopsis sp*. (MIC: 500 μg/ml), *B. cinerea*, *T. rubrum*, *C. albicans*, *A. niger*. (MIC: 1000 μg/ml).

**Table 4:** **Minimum inhibitory concentration of the** ***Streptomyces lavendulae*** **strain SCA5 ethyl acetate extract**

**Microbes**		**Ketoconazole**
*Aspergillus flavus*	>31.25 μg/ml	>12.5 μg/ml
*Aspergillus niger*	>1000 μg/ml	>12.5 μg/ml
*Botrytis cinerea*	>1000 μg/ml	>6.25 μg/ml
*Candida albicans*	>1000 μg/ml	>6.25 μg/ml
*Candida.krusei*	>500 μg/ml	>12.5 μg/ml
*Candida parapsilosis*	>500 μg/ml	>12.5 μg/ml
*Malassesia pachydermatis*	>250 μg/ml	>25 μg/ml
*Scopulariopsis sp.*	>1000 μg/ml	>12.5 μg/ml
*Trichophyton mentagrophytes*	>250 μg/ml	>12.5 μg/ml
*Trichophyton rubrum*	>1000 μg/ml	>12.5 μg/ml
**Gram positive bacteria**		**Streptomycin**
*Staphylococcus aureus*	>500 μg/ml	>6.25 μg/ml
*Micrococcus luteus*	>125 μg/ml	>6.25 μg/ml
*Bacillus subtilis*	>500 μg/ml	>6.25 μg/ml
*Staphylococcus epidermidis*	>500 μg/ml	>6.25 μg/ml
MRSA	>250 μg/ml	>25 μg/ml
**Gram negative bacteria**		
*Klebsiella pneumonia*	>500 μg/ml	
*Enterobacter aerogens*	>1000 μg/ml	>25 μg/ml
*Vibrio parahaemolyticus*	>1000 μg/ml	>25 μg/ml
*Yersinia enterocolitica*	>1000 μg/ml	>6.25 μg/ml
*Salmonella typhimurium*	>250 μg/ml	>30 μg/ml
*Shigella flexneri*	>125 μg/ml	>30 μg/ml
*Proteus vulgaris*	>1000 μg/ml	>6.25 μg/ml
*Salmonella typhi-B*	>250 μg/ml	>6.25 μg/ml

Ketoconazole: Antifungal Agent.

Streptomycin: Antibacterial agent.

### Antioxidant properties

#### Inhibition of DPPH radical

**Figure 2:**
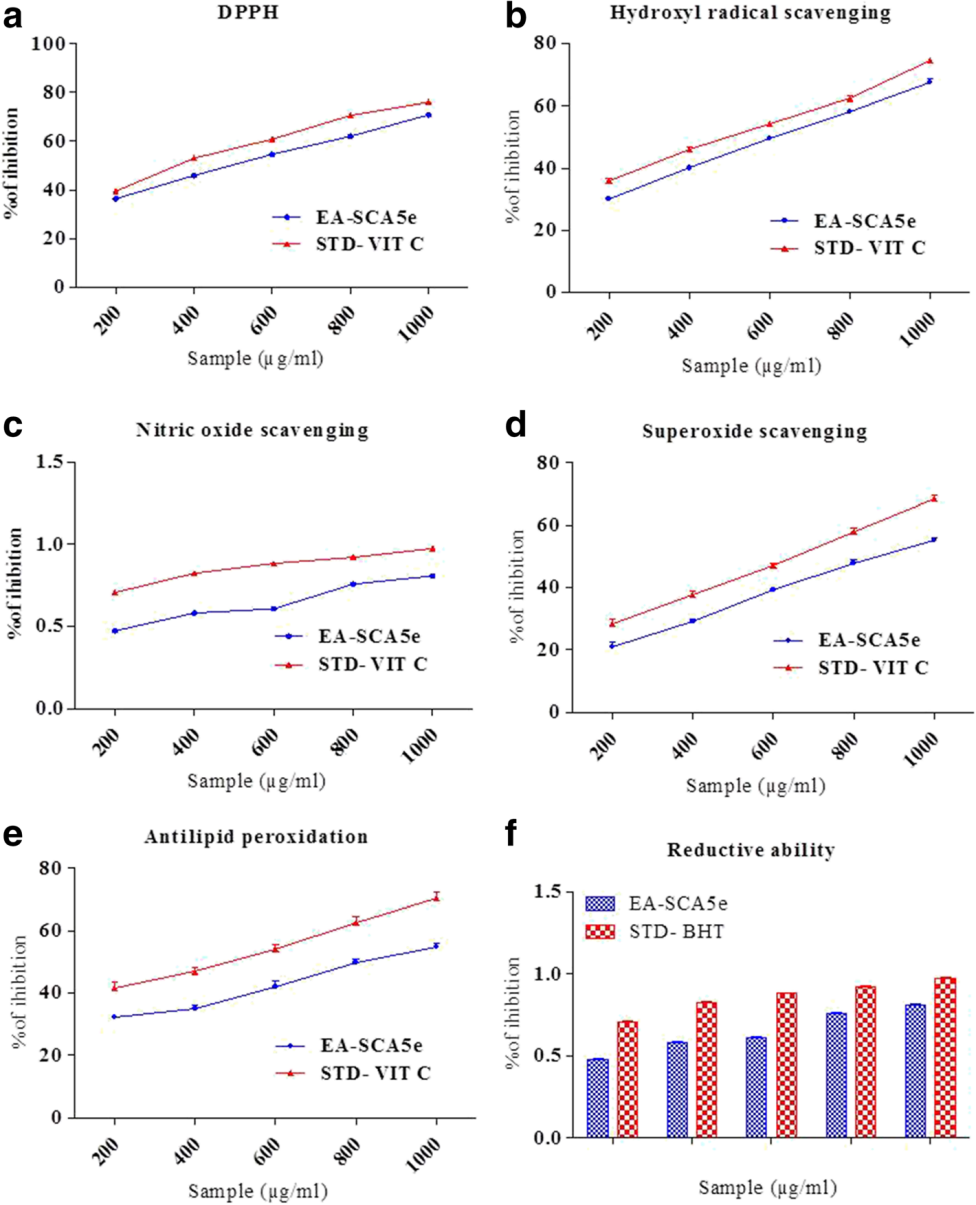
**Antioxidant activity. a**. DPPH radical scavenging activity of different concentrations (200–1000 μg/ml) of EA-SCA5 and Vitamin C. Each value presents the mean ± SEM of triplicate experiments. **b**. Hydroxyl radical scavenging activity of different concentrations (200–1000 μg/ml) of EA-SCA5 and Vitamin **C**. Each value presents the mean ± SEM of triplicate experiments. **c**. Nitric oxide radical scavenging activity of different concentrations (200–1000 μg/ml) of EA-SCA5 and Vitamin C. Each value presents the mean ± SEM of triplicate experiments. **d**. Superoxide anion radical-scavenging activity of different concentrations (200–1000 μg/ml) of EA-SCA5 and Vitamin C. Each value presents the mean ± SEM of triplicate experiments. **e**. Lipid peroxidation inhibition of different concentrations (200–1000 μg/ml) of EA-SCA5 and Vitamin C. Each value presents the mean ± SEM of triplicate experiments. **f**. Reducing power determination of different concentrations (200–1000 μg/ml) of EA-SCA5 and BHT. Each value presents the mean ± SEM of triplicate experiments.

The EA-SCA5 exhibited significant dose dependent inhibition of DPPH activity, with a 50% inhibition (IC_50_) at a concentration of 507.61 ± 0.66 μg/ml. The results are presented in Figure 2A. The IC_50_ value for vitamin C was 382.09 ± 0.67 μg /ml.

### Hydroxyl radical scavenging assay

The results for hydroxyl radical scavenging assay are shown in Figure 2B. The concentrations for 50% inhibition were found to be IC_50_ 617.84 ± 0.57 μg/ml), and 501.49 ± 0.67 μg /ml for the EA-SCA5 and vitamin C, respectively.

### Nitric oxide radical inhibition assay

The scavenging of nitric oxide by the extract was increased in a dose-dependent manner as illustrated in Figure 2C. At the concentration of 730.92 ± 0.81 μg/ml of EA-SCA5, 50% of nitric oxide was scavenged. The IC_50_ value for vitamin C was 419.33 ± 0.67 μg /ml.

### Superoxide

Figure 2D shows the superoxide radical scavenging capacity of EA-SCA5. EA-SCA5 demonstrated a dose–response inhibition of the superoxide anion radicals. EA-SCA5 exhibited superoxide anion radical scavenging activity at all the concentrations. It showed 50% inhibition at 864.71 ± 1.15 μg/ml. The IC_50_ value for vitamin C was 641.76 ± 1.09 μg /ml.

### Lipid peroxidation assay

Activity of EA-SCA5 on lipid peroxidation is shown in Figure 2E. The extract showed inhibition of peroxidation at all the concentrations; it showed 50% inhibition at 838.83 ± 1.18 μg/ml. The IC_50_ value for vitamin C was 458.90 ± 1.26 μg/ml.

### Reducing power

Figure 2F shows the reductive capabilities of the extract compared to the standard butylated hydroxyl toluene. The reducing power of EA-SCA5 increased with increasing quantity of the sample.

### Cytotoxicity property

**Figure 3:**
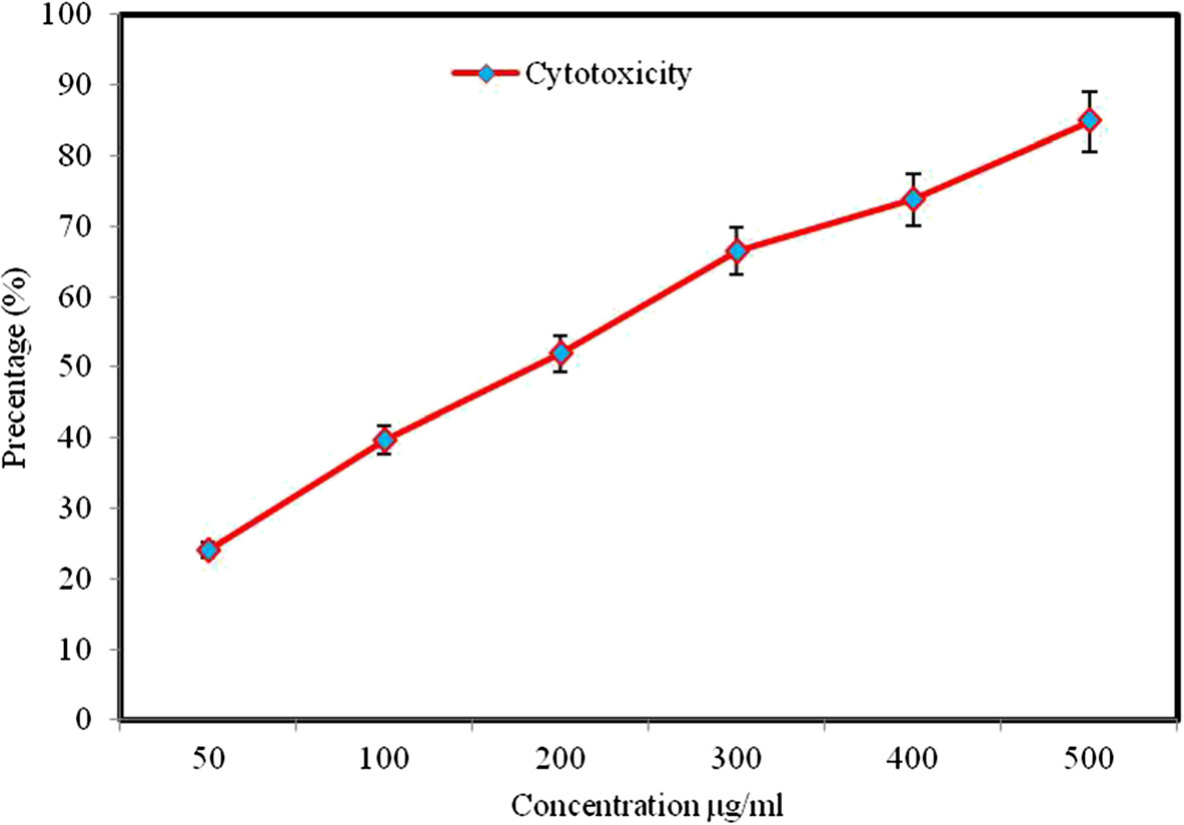
**Cytotoxic effects of EA-SCA5 on cancer cell line (A549).** Data are mean ± SD of three independent experiments with each experiment conducted in triplicate.

EA-SCA5 showed cytotoxic activity *in vitro* against A549 lung adenocarcinoma cancer cell line. It showed 84.9% activity at the dose of 500 μg/ml with IC_50_ value of 51.9% at 200 μg/ml (Figure 3). All the concentrations used in the experiment decreased cell viability significantly (P < 0.05) in a concentration-dependent manner.

### TLC analysis

**Figure 4:**
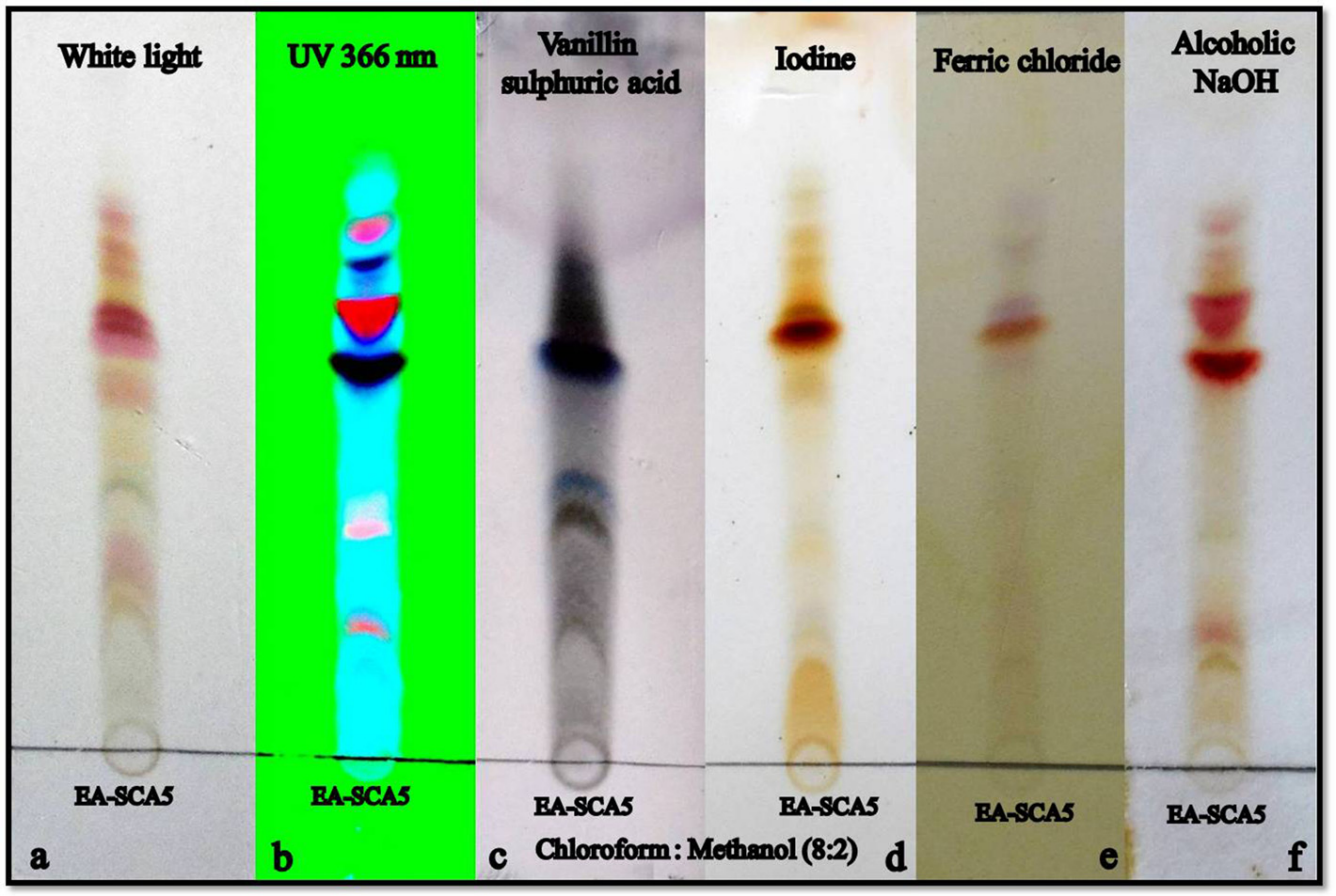
**TLC profile of the active crude from soil** ***Streptomyces lavendulae*** **strain SCA5. a**. Viewed under normal white light. **b**. Viewed under UV 366 nm. **c**. Derivatization with 10% alcoholic sulphuric acid. **d**. Exposure to iodine vapour. **e**. 0.01% ferric chloride. **f**. 0.01% alcoholic NaOH.

TLC profile of the active ethyl acetate extract is given in Figure 4 with various methods of visualization. As shown in Figures 4C and D the extract contained phenol and quinone.

### GC-MS analysis

**Figure 5:**
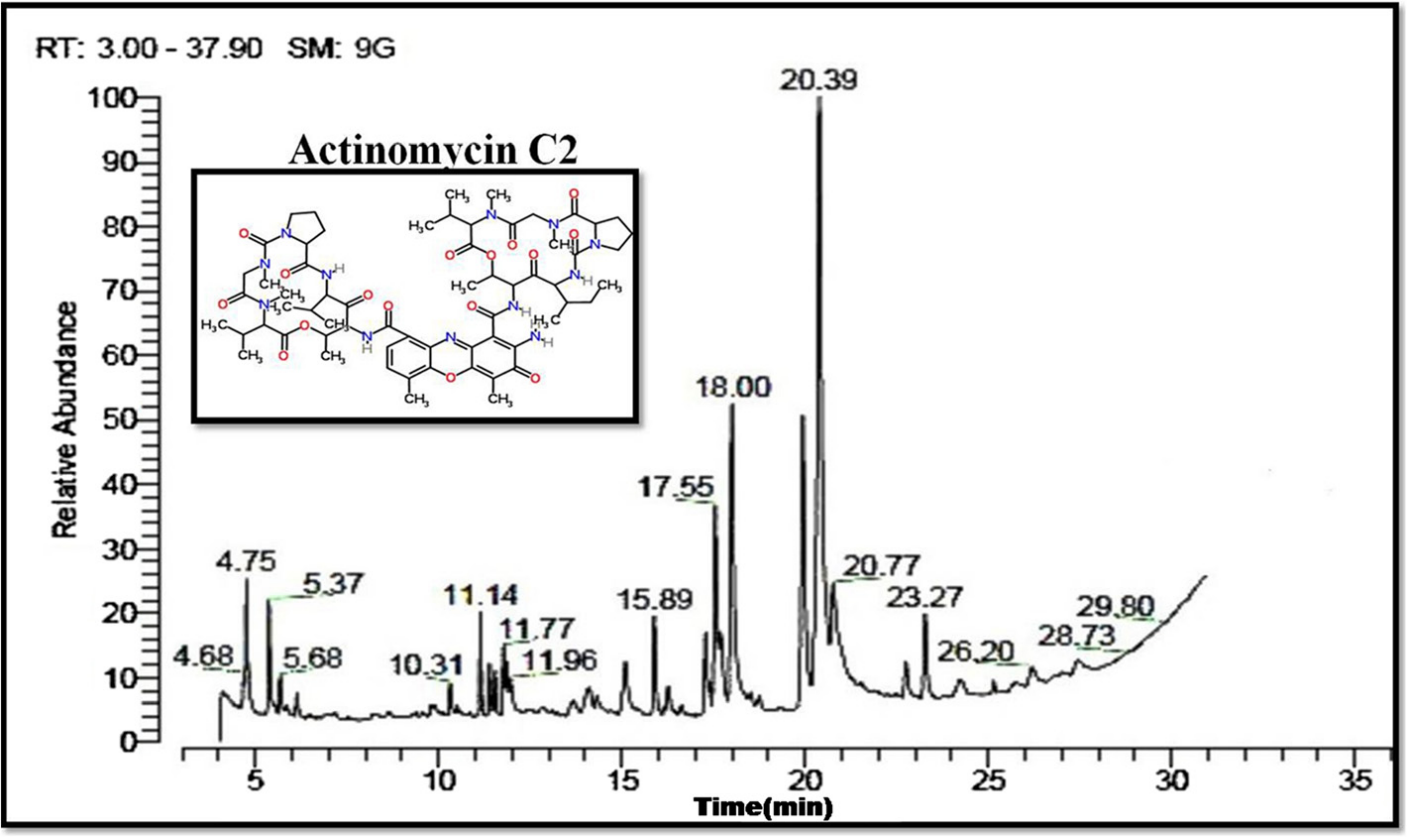
**GC-MS analysis of active crude from soil** ***Streptomyces lavendulae*** **strain SCA5.**

The chemical composition of bioactive extract of EA-SCA5 was investigated using GC-MS analysis. On comparison of the mass spectra of the constituents with the NIST library, eleven compounds were identified by retention time, molecular weight and molecular formula as mentioned in Table 5 and Figure 5. Of the eleven compounds identified, the most important compound was actinomycin C2 which constituted 7.12%.

**Table 5:** **GC-MS profile of the** ***Streptomyces lavendulae*** **strain SCA5 ethyl acetate extract**

**S.no**	**Retention time**	**Compound name**	**Molecular formula**	**Molecular weight**	**Area %**
1.	4.68	Octaethylene glycol monododecyl ether	C_28_H_58_O_9_	538	0.48
2.	4.75	2(3H)Furanone,5acetyldihydro	C_6_H_8_O_3_	128	2.62
3.	5.37	1,4Dioxane2,5dione,3,6dimethyl	C_6_H_8_O_4_	144	2.24
4.	10.31	Hexanoic acid, 2phenylethyl ester	C_14_H_20_O_2_	220	0.59
5.	11.77	2,4Dimethyl3pentanol acetate	C_9_H_18_O_2_	158	1.06
6.	11.96	Hexadecanoic acid, 1(hydroxymethyl)1,2ethanediyl ester	C_35_H_68_O_5_	568	0.86
7.	14.31,16.27,17.28,17.68, 27.47	Actinomycin C2	C_63_H_88_N_12_O_16_	1268	7.12
8.	17.55, 18.00	Hexadecanoic acid	C_16_H_32_O_2_	256	14.75
9.	20.39	Oleic Acid	C_18_H_34_O_2_	282	26.46
10.	20.77	2,5Piperazinedione,3,6bis(2methylpropyl)	C_12_H_22_N_2_O_2_	226	8.42
11.	22.75,23.27	Ergotaman	C_33_H_35_N_5_O_5_	581	3.56

## Discussion

The resistance of numerous pathogenic bacteria and fungi to commonly used bioactive secondary metabolites necessitates the search for new antifungal and antibacterial molecules to combat these pathogens. Secondary metabolites produced by microbes continue to attract the attention, due to their sophisticated chemical structures and highly specific biological activities. Filamentous soil bacteria belonging to the genus *Streptomyces* are rich sources of a high number of bioactive natural products with biological activity; they are extensively used in pharmaceutical and agrochemical industries. These bacteria produce about 75% of commercially and medically useful antibiotics [30]. Newer or rare soil actinomycetes are good sources of potent molecules. 37 actinomycetes were isolated from soil samples collected from Vengodu (agricultural field); among these isolates SCA5 exhibited good antimicrobial activity. SCA5 was identified as *Streptomyces lavendulae*. It had been found that the majority of the soil isolates produced aerial coiled mycelia and the spores were arranged in chains [31]. *Actinomyces* are useful biological tools in the production of antimicrobials against bacteria and fungi [32]. *Streptomyces lavendulae* strain SCA5 showed good antimicrobial activity in solid medium and also in fermented broth. Our results indicated that the antimicrobial metabolites were extracellular. Most of the secondary metabolites and antibiotics are extracellular in nature and extracellular products of actinomycetes show potent antimicrobial activities [33]. From the results obtained, it appeared that the antimicrobial action of EA-SCA5 was more pronounced on both Gram positive and Gram negative bacteria and fungi. These results are consistent with previous reports of Saravana kumar et al. [34] and Vijayakumar et al. [35]. In this study, EA-SCA5 was investigated for the scavenging abilities on DPPH, hydroxyl radicals, nitric oxide radical, superoxide and lipid peroxidation. The presence of good amount of phenolics as shown by Folin–Ciocalteau method might have contributed to its antioxidant potential. DPPH is a useful reagent to evaluate the free radical scavenging ability of the hydrogen donating antioxidant, which can transfer hydrogen atoms or electrons to DPPH radicals [36]. EA-SCA5 was able to reduce the stable radical DPPH to the yellow-colored diphenylpicrylhydrazine. Hydroxyl radical is one of the reactive oxygen species generated in the body, and removing hydroxyl radicals is important for antioxidant defence in living cell systems [37]. The nitric oxides radical inhibition study showed that EA-SCA5 was a potent scavenger of nitric oxide. EA-SCA5 inhibited nitrite formation by competing with oxygen to react with nitric oxide directly and also to inhibit its synthesis. Superoxide anions are precursors to active free radicals that have potential for reacting with biological macromolecules and thereby inducing tissue damage [38]. Antioxidants are able to inhibit the blue NBT formation [39]. The inhibition of superoxide radical by EA-SCA5 was lower than vitamin C. In our study, the inhibition of b-carotene bleaching by EA-SCA5 was lower than the standard for measurement of reductive ability. The reducing power increased with increasing concentration of the EA-SCA5. The reducing capacity of a compound may serve as a significant indicator of its potential antioxidant activity [40]. The studies on soil actinomycetes with respect to cytotoxic activity are very limited in the Indian sub-continent. EA-SCA5 showed good activity against A549 lung adenocarcinoma cancer cell. Chandrananimycins, isolated from marine *Actinomadura* spp. MO48 has been shown to exhibit antibacterial, antifungal and anticancer activity [41]. Eleven compounds were identified from the GC-MS analysis of EA-SCA5. These might be responsible for the antimicrobial, antioxidant and cytotoxity activities. Similar results were reported by Joseph Selvin et al. [42]. The compounds such as 2(3H)Furanone, 5acetyldihydro, 1,4 Dioxane, 2, 5 dione, 3, 6 dimethyl, Hexanoicacid, 2phenylethylester, 2, 4 Dimethyl3pentanolacetate, ActinomycinC2, 2, 5 Piperazinedione, 3, 6 bis(2methylpropyl), Ergotaman were reported to possess activity. The constituents such as Octaethylene glycol monododecyl ether; Hexadecanoic acid and Hexadecanoic acid are straight chain hydrocarbons or alcohols, which are known to be inactive. Oleic acid is used as an excipient in pharmaceuticals and as an emulsifying or solubilising agent in aerosol products [43]. The major compound actinomycin C2 has been reported to have antimicrobial [44], antioxidant [45] and anti-cancerous property including Gestational trophoblastic neoplasia [46], Wilms’ tumor [47], Rhabdomyosarcoma [48], Ewing’s sarcoma [49], Malignant hydatidiform mole [50].

## Conclusion

The ethyl acetate extract of *Streptomyces lavendulae* strain SCA5 displayed significant biological activities against tested Gram positive and Gram negative bacterial pathogens and filamentous fungal pathogens. It also exhibited antioxidant and cytotoxic activity against carcinoma cell lines *in vitro.* GC-MS showed the presences of actinomycin C2 (7.12%) which may be the active principle.

## Additional file

Additional file 1:
**Radical scavenging activity of di fferent concentrations (200-1000 μg/ml) of EA-SCA5e and standards (Vitamin C and Butylated hydroxytoluene BHT).**
