# Supplementary material for: In vitro antimicrobial, antioxidant and cytotoxic properties of Streptomyces lavendulae strain SCA5
Source: BMC Microbiol. 2014 Nov 30;14:291. doi: 10.1186/s12866-014-0291-6 (PMC4265329; doi:10.1186/s12866-014-0291-6)

**Radical scavenging activity of different concentrations (200-1000 μg/ml) of EA-SCA5e and standards (Vitamin C and Butylated hydroxytoluene BHT)**

**Table: 1 DPPH radical scavenging activity**

| **Concentration** | **EA-SCA5e** | | **STD- VIT C** | |
| --- | --- | --- | --- | --- |
| **(in %) ± SD** | | | | |
| 200 µg/ml | 36.347520 | 0.012342 | 39.494680 | 0.467006 |
| 400 µg/ml | 45.966310 | 0.003606 | 53.058510 | 0.427467 |
| 600 µg/ml | 54.609930 | 0.008185 | 60.859930 | 0.905167 |
| 800 µg/ml | 62.056740 | 0.003055 | 70.656030 | 0.732390 |
| 1000 µg/ml | 70.877660 | 0.006245 | 76.063830 | 0.531915 |

**Table: 2 Hydroxyl radical scavenging activity**

| **Concentration** | **EA-SCA5e** | | **STD- VIT C** | |
| --- | --- | --- | --- | --- |
| **(in %) ± SD** | | | | |
| 200 µg/ml | 30.241250 | 0.655362 | 35.881750 | 0.9060341 |
| 400 µg/ml | 40.197080 | 0.561424 | 45.973500 | 0.8343887 |
| 600 µg/ml | 49.541280 | 0.407747 | 54.468230 | 0.523099 |
| 800 µg/ml | 58.273870 | 0.751388 | 62.351340 | 0.9249513 |
| 1000 µg/ml | 67.753990 | 0.866962 | 74.549780 | 0.5796372 |

**Table: 3 Nitric oxide radical scavenging activity**

| **Concentration** | **EA-SCA5e** | | **STD- VIT C** | |
| --- | --- | --- | --- | --- |
| **(in %) ± SD** | | | | |
| 200 µg/ml | 0.4726667 | 0.012342 | 0.706667 | 0.005132 |
| 400 µg/ml | 0.582000 | 0.003606 | 0.824667 | 0.007638 |
| 600 µg/ml | 0.608000 | 0.008185 | 0.884000 | 0.003000 |
| 800 µg/ml | 0.7583333 | 0.003055 | 0.921333 | 0.005859 |
| 1000 µg/ml | 0.808000 | 0.006245 | 0.975000 | 0.002000 |

**Table: 4 Superoxide anion radical-scavenging activity**

| **Concentration** | **EA-SCA5e** | | **STD- VIT C** | |
| --- | --- | --- | --- | --- |
| **(in %) ± SD** | | | | |
| 200 µg/ml | 21.070230 | 1.368788 | 28.483840 | 1.517353 |
| 400 µg/ml | 29.152730 | 0.858127 | 37.848380 | 1.174543 |
| 600 µg/ml | 39.241920 | 0.754055 | 46.989970 | 0.9310642 |
| 800 µg/ml | 47.770340 | 1.392419 | 57.971010 | 1.255109 |
| 1000 µg/ml | 55.295430 | 0.920998 | 68.338910 | 1.517353 |

**Table: 5 Lipid peroxidation inhibition**

| **Concentration** | **EA-SCA5e** | | **STD- VIT C** | |
| --- | --- | --- | --- | --- |
| **(in %) ± SD** | | | | |
| 200 µg/ml | 32.234430 | 0.634451 | 41.758240 | 1.903352 |
| 400 µg/ml | 35.164840 | 1.098901 | 46.886450 | 1.268902 |
| 600 µg/ml | 42.124540 | 1.678599 | 54.212450 | 1.268902 |
| 800 µg/ml | 49.816850 | 1.268902 | 62.637360 | 1.903352 |
| 1000 µg/ml | 54.945050 | 1.098901 | 70.329670 | 2.197802 |

**Table: 6 Reducing power determination**

| **Concentration** | **EA-SCA5e** | | **STD- BHT** | |
| --- | --- | --- | --- | --- |
| **(in %) ± SD** | | | | |
| 200 µg/ml | 0.4726667 | 0.012342 | 0.706667 | 0.005132 |
| 400 µg/ml | 0.582000 | 0.003606 | 0.824667 | 0.007638 |
| 600 µg/ml | 0.608000 | 0.008185 | 0.884000 | 0.003000 |
| 800 µg/ml | 0.7583333 | 0.003055 | 0.921333 | 0.005859 |
| 1000 µg/ml | 0.808000 | 0.006245 | 0.975000 | 0.002000 |

**Figure: Phylogenetic tree of *Streptomyces lavendulae* strain SCA5 (KC315780) constructed using the neighbor-joining method with the aid of MEGA 4.1 program**


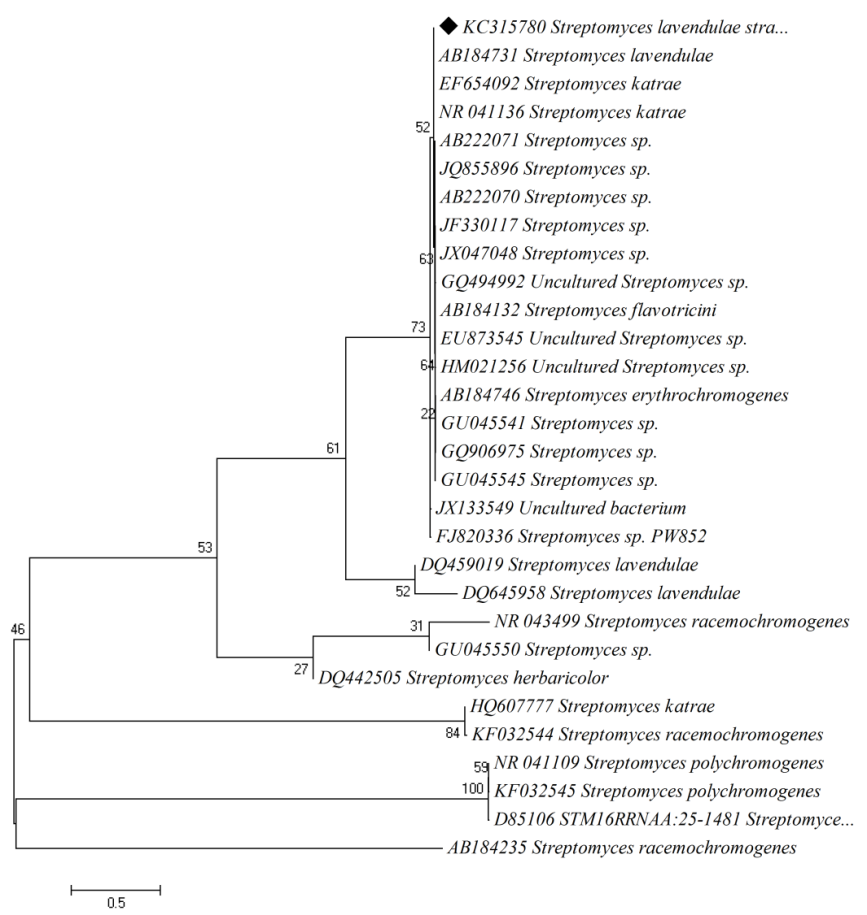

Supplement: Additional file 1: — Radical scavenging activity of di fferent concentrations (200-1000 μg/ml) of EA-SCA5e and standards (Vitamin C and Butylated hydroxytoluene BHT). [file 12866_2014_291_MOESM1_ESM.docx]
